# Ankle-Brachial Index: A Simple Way to Predict Mortality among Patients on Hemodialysis - A Prospective Study

**DOI:** 10.1371/journal.pone.0042290

**Published:** 2012-07-30

**Authors:** Zaida Noemy Cabrera Jimenez, Benedito Jorge Pereira, João Egidio Romão, Sonia Cristina da Silva Makida, Hugo Abensur, Rosa Maria Affonso Moyses, Rosilene Motta Elias

**Affiliations:** Renal Division, Internal Medicine, Hospital das Clínicas, University of São Paulo School of Medicine, São Paulo, Brazil; University of Sao Paulo Medical School, Brazil

## Abstract

**Background:**

Ankle-brachial index (ABI) can access peripheral artery disease and predict mortality in prevalent patients on hemodialysis. However, ABI has not yet been tested in incident patients, who present significant mortality. Typically, ABI is measured by Doppler, which is not always available, limiting its use in most patients. We therefore hypothesized that ABI, evaluated by a simplified method, can predict mortality in an incident hemodialysis population.

**Methodology/Principal Findings:**

We studied 119 patients with ESRD who had started hemodialysis three times weekly. ABI was calculated by using two oscillometric blood pressure devices simultaneously. Patients were followed until death or the end of the study. ABI was categorized in two groups normal (0.9–1.3) or abnormal (<0.9 and >1.3). There were 33 deaths during a median follow-up of 12 months (from 3 to 24 months). Age (1 year) (hazard of ratio, 1.026; p = 0.014) and ABI abnormal (hazard ratio, 3.664; p = 0.001) were independently related to mortality in a multiple regression analysis.

**Conclusions:**

An easy and inexpensive technique to measure ABI was tested and showed to be significant in predicting mortality. Both low and high ABI were associated to mortality in incident patients on hemodialysis. This technique allows nephrologists to identify high-risk patients and gives the opportunity of early intervention that could alter the natural progression of this population.

## Introduction

Despite significant technological advances, mortality among patients on hemodialysis remains inexcusably high, particularly in the first year of therapy [1].

One way to potentially reduce mortality is to alert medical staff to patients at increased risk of death in order to facilitate timely diagnostic and therapeutic interventions.

Hemodialysis patients are at an increased risk for atherosclerotic disorders, including peripheral arterial disease (PAD) [2]. PAD is a strong predictor for all-cause and cardiovascular mortality in hemodialysis patients [3], [4].

Ankle-brachial index (ABI) is a simple, noninvasive, and reliable test for PAD screening. Usually ABI is classified as low (<0.9), normal (0.9–1.3) and high (>1.3). Both low and high ABI are correlated to high mortality in patients on hemodialysis [5]. Low ABI is related to PAD, and high ABI is caused by no compressible arteries and vascular calcification. Therefore, an abnormal ABI value is a significant predictor of mortality among patients on hemodialysis [4], [6].

ABI was already identified as a predictor of mortality among prevalent patients on hemodialysis. Incident patients on hemodialysis are even at higher risk. The annual number of patients beginning dialysis has grown, mostly because of the growing prevalence of hypertension and diabetes, the main underlying cause of kidney disease [7], [8]. Most analyses of survival on dialysis exclude the first 90-day period. There have been minimal improvements in mortality rate during the first year, over the past decade, even without including the initial 90 days [1], [7]. ABI could be an easy way to identify patients at the highest risk.

We recently tested measuring ABI by using two oscillometric blood pressure devices simultaneously [9], simulating an ABI-form device.

The results demonstrated good reproducibility and low intra-observer variability. The ABI was extensively tested in three consecutive dialysis sessions, as well as pre and post dialysis. The question that remained was if this technique could be significant in predicting mortality.

We therefore tested the general hypothesis that ABI measured by two oscillometric blood pressure devices can predict mortality in an incident hemodialysis population.

## Methods

### Subjects

**Table 1:** Characteristics of the study population.

Variables	N = 119
Age, years	53.1±18.8
Male, n (%)	82 (68.9)
Etiology of ESRD, n (%)	
Hypertension	35 (29.4)
Diabetes	44 (37.0)
Glomerulonephritis	18 (15.1)
Others	22 (18.5)
Hypertension, n (%)	118 (99.1)
Diabetes mellitus, n (%)	49 (47.2)
Family history of CV disease, n (%)	62 (52.1)
Smoking, ever vs. never, n (%)	36 (30.2)
Sedentary habits, n (%)	92 (77.3)
Ankle-brachial index	1.16±0.23
Systolic Blood Pressure, mmHg	149.4±23.8
Diastolic Blood Pressure, mmHg	85.9±17.2
Albumin level, g/dl	3.4±0.7
Phosphorus level, mg/dl	5.8±2.2
Calcium corrected, mg/dl	9.0±1.1
Calcium x Phosphorus, mg/dl	48.3±18.3
Total cholesterol, mg/dl	167.9±44.9
Hemoglobin, g/dl	9.3±1.7
C-reactive protein, mg/L	35.9±48.9
Parathyroid hormone, pg/ml	372.7±484.2

CV, cardiovascular; Calcium corrected  =  calcium + (4-albumin) ×0.8.

Values are expressed as mean ± SD or n (percentage).

Patients were enrolled from January 2008 to January 2010. Inclusion criteria were patients with end-stage renal disease (ESRD) at least 18 years of age undergoing their first month of conventional hemodialysis three times weekly in the University of Sao Paulo School of Medicine’s Hospital das Clinicas. This was a prospective observational study.

We excluded anyone who had been treated for infection, had bilateral amputation, bilateral fistula or had atrial fibrillation. Prior to ABI measurement, demographic characteristics, and medical history were recorded. Biochemical data were measured within 2 weeks of ABI measurement and dialysis initiation.

### ABI Measurement

The measurements were done by using two oscillometric devices (Omron Corp 705 CP Corp, Tokyo, Japan) simultaneously to measure blood pressure in the upper and lower extremities. The pre dialysis ABI was used in this new technique which was already intra-observer validated and described [9]. Briefly, measurements were done pre dialysis, with the patient at rest in the supine position, after 5 minutes of the beginning of the session. The protocol was performed by the same examiner in all situations. During the study, only raw blood pressure values were recorded, and stored on a database for later ABI calculation. Our technique showed that ABI can be measured by two oscillometric devices at the same time, with good reproductibility and low intra-observer variation.

### Follow-up

The outcome of this study was all-cause mortality. Patients were censored in case of renal transplantation or recovery of renal function. Vital status was ascertained by telephone contact. The censor date was 31st January 2010. Date of death was verified by contacting relatives and/or the staff of the dialysis clinic. Survival time was defined as the time of the baseline (first day of ABI measurement) to the date of death or end of follow-up.

### Ethic Statements

The protocol was approved by the Research Ethics Boards of the University of Sao Paulo School of Medicine, and all subjects provided written informed consent before participation.

### Data Analysis

Differences between continuous variables and categorical variables were calculated using Student t test for normally distributed continuous variables and by Mann-Whitney U test for abnormally distributed variables. The χ2 or Fisher exact test was used to compare nominal variables. To compare the 3 groups according to ABI <0.9, from 0.9 to 1.3 and >1.3, one-way analysis of variance was applied, with Tukey post-hoc test.

Mortality was the outcome of the follow-up. The relation of ABI categories and all-cause mortality was analyzed by Kaplan-Meier survival plots and log-rank test. A stepwise multivariate cox regression was undertaken to identify the relative risk of all-cause mortality (entry threshold, P<0.05; removal threshold, P<0.10) with the significant candidate variables (age, diabetes, calcium x phosphorus product, parathyroid hormone and C-reactive protein). A lower level of the –2 log-likelihood indicates a better model fit. Data are presented as means ±SD unless indicated otherwise. A P-value <0.05 was considered significant. Analyses were performed with the use of SPSS 17.0.1 (SPSS Inc., Chicago, Ill).

## Results

### Subjects

Out of 123 consecutive ESRD patients, 4 lost the follow-up. Therefore, 119 patients were studied (82 men and 37 women), whose baseline characteristics were shown in Table 1. Diabetes, hypertension and glomerulonephritis accounted for 80% of the underlying causes of renal disease. The majority of the patients had a family history of cardiovascular disease (51.2%), 30.2% were smokers, 47.2% had diabetes, hypertension was found in almost 100% of the patients, and 77.3% presented sedentary behavior, which configure in a high cardiovascular risk population. Anemia, altered bone mineral metabolism, and inflammation (expressed by C-reactive protein) were also observed.

**Figure 1:**
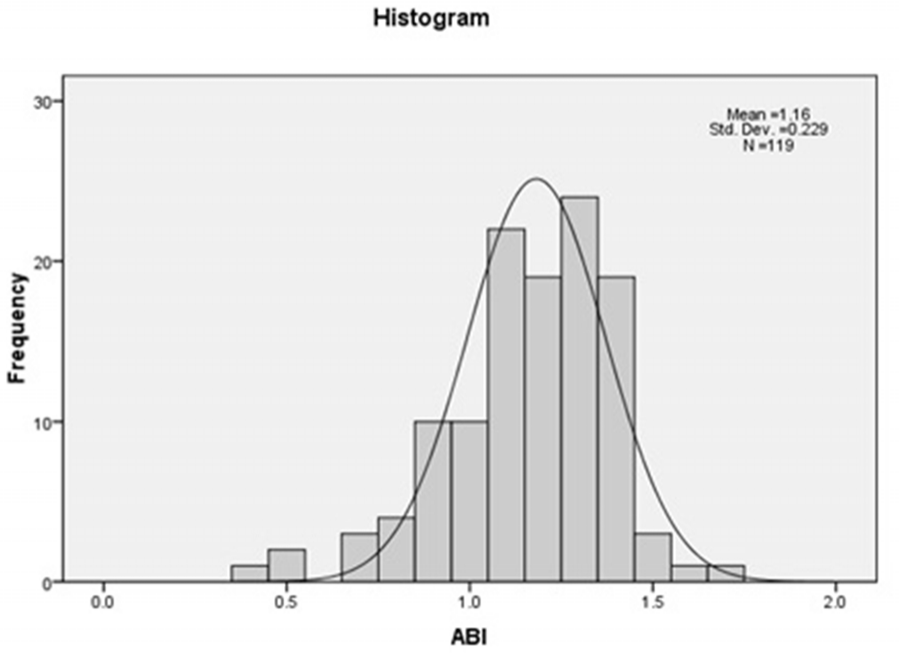
Frequency distribution of baseline ankle-brachial index (ABI).

Characteristics of the study population regarding ABI categories (normal 0.9–1.3, low <0.9, and high >1.3 are presented in Table 2. Distribution of observed ABI at the baseline is shown in Figure 1. The significant differences between these 3 groups were age (p = 0.011), albumin (p = 0.040) and hemoglobin (p = 0.010). Cardiovascular risk factors observed at the baseline were well distributed among the three groups.

**Table 2:** Characteristics of the study population according to ABI.

Variable	ABI			
	<0.9 N = 12	0.9–1.3 N = 73	>1.3 N = 34	p
Age, years	68.8±16.6*	53.7±17.9	42.3±18.2	0.001
Male sex, n (%)	7 (58.3)	49 (67.1)	26 (76.5)	0.440
Diabetes mellitus, n (%)	5 (41.7)	29 (39.7)	15 (44.1)	0.911
Family history of CV disease, n (%)	6 (50)	41 (56.2)	15 (44.1)	0.503
Smoking, ever vs. never, n (%)	4 (33)	23 (31.5)	9 (26.5)	0.844
ABI	0.68±0.16*	1.13±0.11	1.40±0.08*	0.0001
Albumin level, g/dl	3.4±0.9	3.5±0.6	3.1±0.8*	0.040
Calcium x Phosphorus, mg^2^/dl^2^	47.7±22.0	49.7±18.6	45.6±16.6	0.564
Total cholesterol, mg/dl	162.4±36.8	168.3±46.9	169.1±43.9	0.903
Hemoglobin, g/dl	9.9±2,1	9.5±1.6	8.6±1.5*	0.010
C-reactive protein, mg/l	55.0±69.8	31.0±41.3	39.6±54.9	0.260
Parathyroid hormone, pg/ml	463.6±302.1	397.7±555.7	286.2±360.4	0.448

CV, cardiovascular; ABI, ankle-brachial index; Corrected Calcium  =  calcium + (4-albumin) ×0.8.

Values are expressed as Mean ± SD. *p<0.05 by post-test, comparing to ABI 0.9–1.3.

During the study there were 33 deaths on a median follow-up of 12 months (from 3 to 24 months). Table 3 shows the differences in basal characteristics between the group of patients who died and the group that survived. Patients who died were more likely to be older (p = 0.003), and with lower PTH (p = 0.044). Gender, diabetes, family history of cardiovascular disease, smoking and sedentary habitus, blood pressure, and biochemical variables did not distinguish survivors and non-survivors. The ABI as a continuous variable was virtually identical in survivors and non-survivors (p = 0.998). However, abnormal ABI was more frequent among patients who died than in patients who survived (63.6% vs. 13.9%, p<0.0001).

**Table 3:** Survivors and non-survivors.

Variable	Survivors N = 86	Non-survivors N = 33	p
Age, years	50.0±18.3	61.2±17.8	0.003
Male sex, n (%)	58 (67.4)	27 (81.8)	0.671
Diabetes mellitus, n (%)	31 (36.0)	19 (57.6)	0.161
Family history of CV disease, n (%)	44 (51.2)	19 (57.6)	1
Smoking, ever vs. never, n (%)	26 (30.2)	10 (30.3)	0.830
Sedentary habitus, n (%)	68 (79.1)	26 (78.8)	0.355
ABI	1.16±0.18	1.15±0.32	0.998
Abnormal ABI, %	13.9	63.3	<0.0001
Systolic BP, mmHg	150.5±25.4	148.7±24.0	0.735
Diastolic BP, mmHg	86.9±19.9	81.0±16.9	0.146
Albumin level, g/dl	3.5±0.6	3.2±0.8	0.104
Calcium x Phosphorus, mg^2^/dl^2^	50.2±19.2	43.5±15.1	0.195
Total cholesterol, mg/dl	168.5±40.5	166.3±55.3	0.810
Hemoglobin, g/dl	9.2±1.8	9.4±13	0.612
C-reactive protein, mg/l	29.5±39.6	52.8±65.5	0.071
Parathyroid hormone, pg/ml	412.0±549.1	266.5±203.5	0.044

CV, cardiovascular disease; ABI, ankle-brachial index.

Values are expressed as Mean ± SD.

**Figure 2:**
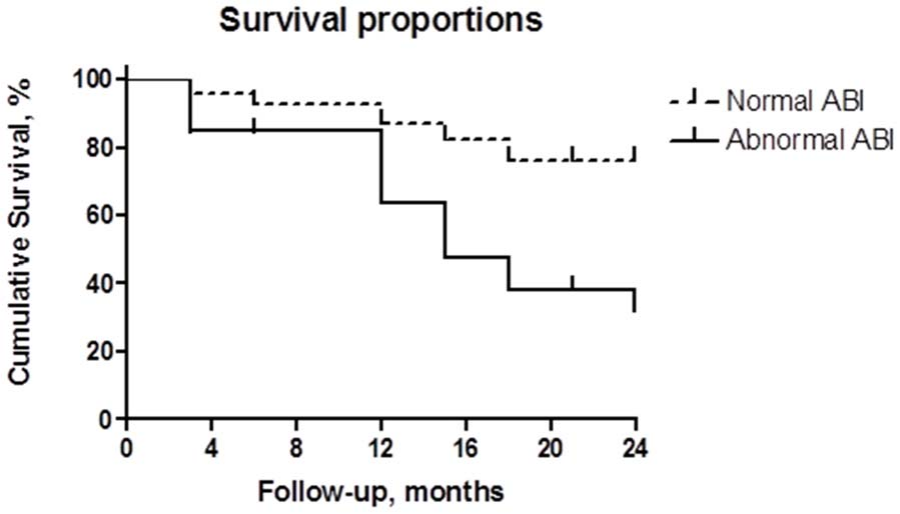
Kaplan-Meier survival curves according to ABI categories: normal (0.9–1.3) or abnormal (<0.9 and >1.3). A lower risk of mortality was observed for normal ABI [superior dashed line]; A higher risk of mortality was observed for abnormal ABI [continuous line]. Log-rank p = 0.0003;

Figure 2 shows an unadjusted Kaplan-Meier survival curve analysis demonstrating that abnormal ABI was associated with greater all-cause mortality. The mean survival time and 95% confidence interval was 21.1±0.8 months (19.6–22.6) for normal ABI, and 16.2±1.2 months (13.8–18.7) for abnormal ABI; log-rank test p = 0.0001.

Upon multivariable Cox regression analyses of all-cause mortality by forward stepwise, the association between abnormal ABI and mortality remained. The HR associated with abnormal ABI was 3.664 times higher than normal ABI (p = 0.001, 95% CI, 1.708–7.861) (Table 4).

**Table 4:** Cox regression Survival Analyses of All-cause Mortality, by Forward Stepwise (Likelihood ratio).

Variable	Hazard Ratio (95% Confidence interval)	*P*
Model 1		*0.001 (entire model)*
Abnormal ABI	3.564 (1.663–7.638)	0.001
Model 2		*0.0001 (entire model)*
Age (1 year)	1.026 (1.005–1.048)	0.014
Abnormal ABI	3.664 (1.708–7.861)	0.001

Initial Log Likelihood function −2 Log likelihood: 255.798.

Variables in the model: age, ABI as categorical (normal and abnormal), diabetes, C-reactive protein, albumin, hemoglobin, and PTH.

## Discussion

Our study has given rise to important findings that provide novel possibilities into the diagnostic and interventions in an incident population on hemodialysis. We found that abnormal baseline ABI can predict mortality in hemodialysis patients. In addition, we accessed ABI by a simple and easy method. These findings suggest that ABI may be measured at patient’s entry on hemodialysis therapy, even in centers without Doppler or an ABI-form device.

Our study population was representative of the total dialysis population in Brazil, according to the Brazilian Society of Nephrology [10], [11] in terms of age, gender distribution and cause of ESRD. The prevalence of diabetes is also similar to the American and European reports [7], [12], [13]. However, all these data refer to the prevalent population and we studied an incident population. This specific population has an even higher risk of mortality. Some reported annual rate of mortality simply does not take into account the first 3 months of hemodialysis. The lack of information in the literature regarding diagnostic and possible therapeutic interventions in this selected population calls for new studies.

The incidence of patients in hemodialysis has increased in Brazil. The estimated number of patients starting treatment in 2010 was 18,972 (incidence rate: 99.5/million) [11].

We demonstrated that our sample size of incident patients on hemodialysis is at a high risk of mortality, with a family history of cardiovascular events, sedentary and smoking habits, presence almost universal of hypertension, diabetes and all dialysis-related biochemical abnormalities also associated to the high mortality risk. Indeed, we observed that 27.7% of the entire population had died after a 2-year period of follow-up.

The present data build on the previous findings showing that ABI is a good tool to detect high risk patients on hemodialysis. However, a mainly prevalent population on hemodialysis was studied, showing that both low and high ABI were associated to high overall and cardiovascular mortality [4], [5], [14], [15].

We could demonstrate similar results of previous studies by using a modified method in measuring ABI. It is important, however, to emphasize, that we had previously tested the intra-observer reproducibility. Based on this, we may suggest the use of this simple technique but we not guarantee applicability to other dialysis population. Different from previous studies, we accessed patients within their first month of dialysis. This approach allows us early recognition of patients at high risk of mortality.

The critical question is if the use of our simple technique in detecting abnormal ABI can eventually improve outcomes. There is no indication whether or not knowing the prognosis will improve outcomes. Possibly, we can at least alert medical staff to implement therapeutic interventions in those patients with abnormal ABI. Nevertheless, the clinical utility of our technique has to be studied rigorously in further studies. These studies should enrol larger sample sizes and evaluate different examiners measuring ABI.

Our study is subject to some limitations. First, it only shows associations between abnormal ABI and mortality in one selected dialysis population; this may not be applicable to all hemodialysis population. Second, the sample size was small. Third, we did not compare our technique to the gold-standard method, Doppler, in order to prove they can be interchangeable, although this was not an objective of the present data. Forth, we had no access to medication in use and dose of dialysis during the follow-up period. Finally, we could not distinguish between overall and cardiovascular cause of mortality, because the lack of accurate information relevant to the deaths of the patients within the study population.

However, our method was extensively tested and validated previously, and although we examined a small sample size, a direct relationship between abnormal ABI and mortality was found.

In conclusion, these data suggest that abnormal ABI was associated with increased mortality among incident patients on hemodialysis. The ABI could be measured by using at the same time two oscillometric blood pressure devices and then, making an easier and more largely applicable method in detecting patients with the highest risk of mortality. Our results also suggest the need for randomized trials to determine whether more aggressive interventions will change the outcomes.
